# Dysregulated CD38 expression on T cells was associated with rapidly progressive interstitial lung disease in anti-melanoma differentiation-associated gene 5 positive dermatomyositis

**DOI:** 10.3389/fimmu.2024.1455944

**Published:** 2024-11-11

**Authors:** Yixue Guo, Hongjiang Liu, Bo Chen, Keyi Zhang, Liye Meng, Lin Yan, Qian Niu, Junlong Zhang, Geng Yin, Yi Li

**Affiliations:** ^1^ Department of Laboratory Medicine, West China Hospital, Sichuan University, Chengdu, China; ^2^ Sichuan Clinical Research Center for Laboratory Medicine, Chengdu, China; ^3^ Clinical Laboratory Medicine Research Center of West China Hospital, Chengdu, China; ^4^ Department of Rheumatology and Immunology, West China Hospital, Sichuan University, Chengdu, China; ^5^ Health Management Center, General Practice Medical Center, West China Hospital, Sichuan University, Chengdu, China

**Keywords:** anti-MDA5 antibody, dermatomyositis, T cells, CD38, interstitial lung disease

## Abstract

**Background:**

Anti-melanoma differentiation-associated gene 5 positive dermatomyositis (MDA5+ DM) is a life-threatening disease due to rapidly progressive interstitial lung disease (RP-ILD). We aimed to investigate the expression profile of T cell subsets in MDA5+ DM patients, seeking for possible disease-causing T cell subsets and potential biomarkers to distinguish ILD, especially RP-ILD patients.

**Methods:**

Peripheral blood T cell subpopulations were immunophenotyped in 24 MDA5+ DM patients and 21 healthy controls (HCs) by flow cytometry. The proportion of T cell subsets and clinical characteristics were analyzed. The quantitative determination of cytokines in the plasma was measured by using a microsphere-based immunofluorescence assaying kit.

**Results:**

The proportion of naïve and CD38+ T cells were much higher, whereas the proportion of central memory T cells were lower in MDA5+ DM patients than in HCs. Notably, the proportion of CD38+CD4+ T cells and CD38+CD8+ T cells on T cells in in RP-ILD group were significantly elevated compared to C-ILD, non-ILD group and HCs. Moreover, serum IFN-α levels were significantly increased in MDA5+ DM patients with RP-ILD. Further, the frequencies of CD38+CD4+ T cells and CD38+CD8+ T cells were positively correlated with IFN-α levels. Finally, ROC analysis indicated that CD38+CD4+ T cells and CD38+CD8+ T cells could be potential biomarkers for predicting ILD/RP-ILD in MDA5+ DM patients.

**Conclusion:**

Dysregulated CD38 expression on T cell subsets was associated with lung involvement, especially RP-ILD in MDA5+ DM patients. CD38+ T cell subsets could be used as potential biomarkers for predicting ILD/RP-ILD in MDA5+ DM patients.

## 1 Introduction

Anti-melanoma differentiation-associated gene 5-positive dermatomyositis (MDA5+ DM) is a unique subtype of idiopathic inflammatory myopathy (IIM), characterized by typical rashes, minimal muscle involvement and an increased risk of interstitial lung disease (ILD) (1, 2). Based on the presence and severity of ILD, MDA5+ DM can be divided into three subtypes: rapidly progressive ILD (RP-ILD), chronic-ILD (C-ILD) and non-ILD (3). These three subgroups each accounted for approximately one-third of the total MDA+ DM patients (4, 5). The susceptibility of MDA5+ DM patients to RP-ILD results in poor prognosis and high mortality (4). Despite receiving simultaneous therapy with corticosteroids and powerful immunosuppressive medications, MDA5+ DM-RP-ILD patients still experienced a high 6-month mortality rate ranging from 50-70% (6, 7). The exact pathogenesis underlying the critically ill MDA5+ DM patients remains to be elucidated.

The pathophysiology of several autoimmune diseases is related to aberrant T cell homeostasis, including systemic lupus erythematosus (SLE) and type 1 diabetes (8, 9). Previous studies have found that periphery T cells significantly changed in MDA5+ DM patients (10, 11). More specifically, the frequencies of CD4+CXCR4+ T cells and ISG15+CD8+ T cells were considered to be promising prognostic biomarkers in MDA5+ DM (12, 13). However, the expression characteristics of T cell subsets in different subtypes of MDA5+ DM are still not fully studied. Additionally, the associations between T cell tolerance breach biomarkers and ILD have not been comprehensively evaluated.

Here, we comprehensively analyzed the characteristics of T cell subsets among different MDA5+ DM subpopulations, aiming to reveal new insights into the relationship between T lymphocytes and pulmonary interstitial lesions and find potential biomarkers for predicting RP-ILD in MDA5+ DM patients.

## 2 Materials and methods

### 2.1 Study subjects

24 MDA5+ DM patients were enrolled in this study, together with 21 healthy controls (HCs) matched for age and gender. All patients were treated in Department of Rheumatology and Immunology, West China Hospital between May 2023 and December 2023, and diagnosed based on the 2017 European League Against Rheumatism/American College of Rheumatology classification criteria for IIMs (14) by professional rheumatologists. ILD was identified based on pulmonary high-resolution computed tomography (HRCT). RP-ILD was defined as a progressive deterioration of interstitial lesions within 3 months based on the radiological assessment of HRCT examination, accompanied by severe dyspnoea symptoms (15, 16). The study was reviewed and approved by the Ethics Committee of West China Hospital (2023–794), and all participants gave informed, written consent.

### 2.2 Data collection

Detailed patient information regarding demographics, clinical and laboratory data were collected. Laboratory data mainly include routine blood and coagulation function tests, biochemistry tests, immunoglobulin levels, complement levels and infection biomarker evaluation.

### 2.3 Flow cytometry

Fresh whole blood specimens were collected in EDTA tubes and used for direct antibody staining. After surface antibody staining, the specimens were treated with BD lysis buffer (BD Biosciences) to eliminate red blood cells. Flow cytometry antibodies used include CD3 (Cat# C41176, APC-A750), CD3 (Cat# C41179, FITC), CD4 (Cat# C41174, PC5.5), CD8 (Cat# C41165, APC), CD25 (Cat# C41200, PE), CD127 (Cat# C41177, PC7), CD27 (PC7), CD45RA (Cat# C41158, FITC), CD28 (Cat# C41182, PE), CD38 (Cat# C41191, APC-A750), and HLA-DR (Cat# C41192, PB450), all from Beckman coulter, USA. The stained cells were acquired in a flow cytometer (DxFLEX, Beckman coulter, USA). Flow cytometry analysis was performed with CytExpert software (Beckman Coulter, USA).

### 2.4 Cytokine analysis

The quantitative determination of cytokines in the plasma was performed by using a multiplex immunoassay (Saiji Biotechnology, Jiangxi, China), which provides a microsphere-based system that allows the simultaneous analysis of multiple cytokines, including interleukin (IL)-1β, IL-2, IL-4, IL-5, IL-6, IL-8, IL-10, IL-12p70, IL-17, interferon (IFN)-α, IFN-γ and tumor necrosis factor (TNF)-α.

### 2.5 Statistical analysis

SPSS21.0 (IBM, USA) was used to analyze the data. Numerical data with normal distribution and non-normal distribution were presented as mean ± standard deviation and median (range), respectively. Statistical differences between continuous variables were determined by student’s t test (between two groups) and a one-way analysis of variance (ANOVA) (between multiple groups). Differences between categorical variables were tested using Pearson’s chi-square test. Potential value of CD38+CD4+ and CD38+CD8+ T cells to predict ILD or RP-ILD in MDA5+ DM patients was evaluated using receiver operating characteristic (ROC) analysis. In addition, the Youden’s index was generated to evaluate the optimized cut-off point and calculate the exact sensitivity and specificity of the variable. A *P* value less than 0.05 was considered to be statistically significant.

## 3 Results

### 3.1 General characteristics

A total of 24 patients with MDA5+ DM and 21 sex- and age-matched HCs were enrolled in the study. The demographics, clinical and laboratory features of the patients were shown in **Table 1**. According to the clinical manifestations of lung involvement, MDA5+ DM patients were classified into three subgroups: RP-ILD (n = 9, 37.5%), chronic-ILD (C-ILD, n = 11, 45.8%) and non-ILD (n = 4, 16.7%). The mean age at disease onset of RP-ILD, C-ILD and non-ILD was 48.22 ± 13.24, 42.82 ± 7.78 and 53.50 ± 12.45 years, respectively. In addition, there were no significant differences in clinical parameters and laboratory indicators between C-ILD and RP-ILD patients. Extensive clinical characteristics and laboratory parameters of these three groups were detailed in **Table 1**.

**Table 1:** Comparison of clinical and laboratory characteristics of MDA5 positive DM patients (with RP-ILD, C-ILD and non-ILD).

Variables	RP-ILD n = 9	C-ILD n = 11	non-ILD n = 4	*P* value*
Demographics				
Female, no. (%)	7 (77.8)	10 (90.9)	3 (75)	0.566
Age at onset, years	48.22±13.24	42.82±7.78	53.50±12.45	0.270
Clinical variables				
Gottron papules, no. (%)	1 (11.1)	2 (18.2)	1 (25)	1.000
Mechanic’s hands, no. (%)	1 (11.1)	1 (9.1)	0 (0)	1.000
V-neck sign, no. (%)	1 (11.1)	3 (27.3)	0 (0)	0.591
Shawl sign, no. (%)	0 (0)	2 (18.2)	0 (0)	0.479
Skin ulceration, no. (%)	2 (22.2)	3 (27.3)	0 (0)	1.000
Myalgia, no. (%)	5 (55.6)	3 (27.3)	1 (25)	0.362
Muscle weakness, no. (%)	2 (22.2)	3 (27.3)	1 (25)	1.000
Cough, no. (%)	5 (55.6)	7 (63.6)	2 (50)	1.000
Breathing difficulty, no. (%)	4 (44.4)	4 (36.4)	1 (25)	1.000
Velcro rales, no. (%)	4 (44.4)	2 (18.2)	1 (25)	0.336
Laboratory features				
Hb, g/L	122.22±19.21	112.09±13.92	131.75±8.85	0.188
Lym, × 10^9^/L	0.81±0.49	0.82±0.41	1.55±0.93	0.967
Alb, g/L	34.87±3.71	36.93±5.06	41.38±4.10	0.323
Glu, mmol/L	5.28±2.42	4.77±1.30	5.07±1.14	0.553
TG, mmol/L	1.63±0.55	2.10±0.77	2.48±1.34	0.147
TC, mmol/L	4.57±1.00	4.52±1.41	5.58±0.77	0.932
HDL-C, mmol/L	1.32±0.69	1.94±3.14	1.41±0.31	0.572
LDL-C, mmol/L	2.71±0.72	2.72±1.13	3.48±0.98	0.973
CK, IU/L	29.44±11.82	66.82±86.82	49.25±32.90	0.218
LDH, IU/L	359.89±85.52	315.91±176.98	312.75±32.26	0.505
HBDH, IU/L	281.44±69.58	241.00±137.12	244.50±26.79	0.433
IgG, g/L	15.28±10.69	12.18±3.51	10.17±2.15	0.400
IgA, mg/L	1793.25±697.81	2292.00±973.30	2817.50±1711.54	0.241
IgM, mg/L	1766.00±1058.39	1369.80±792.21	1380.50±873.92	0.376
C3, g/L	0.94±0.13	0.98±0.16	0.86±0.14	0.673
C4, g/L	0.31±0.12	0.28±0.05	0.22±0.05	0.546
CRP, mg/dL	28.29±38.14	18.68±23.38	2.36±1.31	0.505
ESR, mm/h	57.00±22.63	35.60±27.61	34.00±0.00	0.382
D-dimer, mg/L	3.36±4.32	1.28±1.25	0.16±0.00	0.219

RP-ILD, rapidly progressive interstitial lung disease; C-ILD, chronic interstitial lung disease; Hb, hemoglobin; Lym, lymphocyte count; Alb, albumin; Glu, glucose; TG, triglyceride; TC, total cholesterol; HDL-C, high-density lipoprotein cholesterol; LDL-C, low-density lipoprotein cholesterol; CK, creatine kinase; LDH, lactate dehydrogenase; HBDH, hydroxybutyrate dehydrogenase; IgG, immunoglobulin G; IgA, immunoglobulin A; IgM, immunoglobulin M; C3, complement component 3; C4, complement component 4; CRP, C-reactive protein; ESR, erythrocyte sedimentation rate.

* The P-value here is an indicator used to evaluate whether the difference between the RP-ILD and C-ILD groups is statistically significant.

### 3.2 Expression profile of T cell subsets in peripheral blood of MDA5+ DM patients

**Figure 1:**
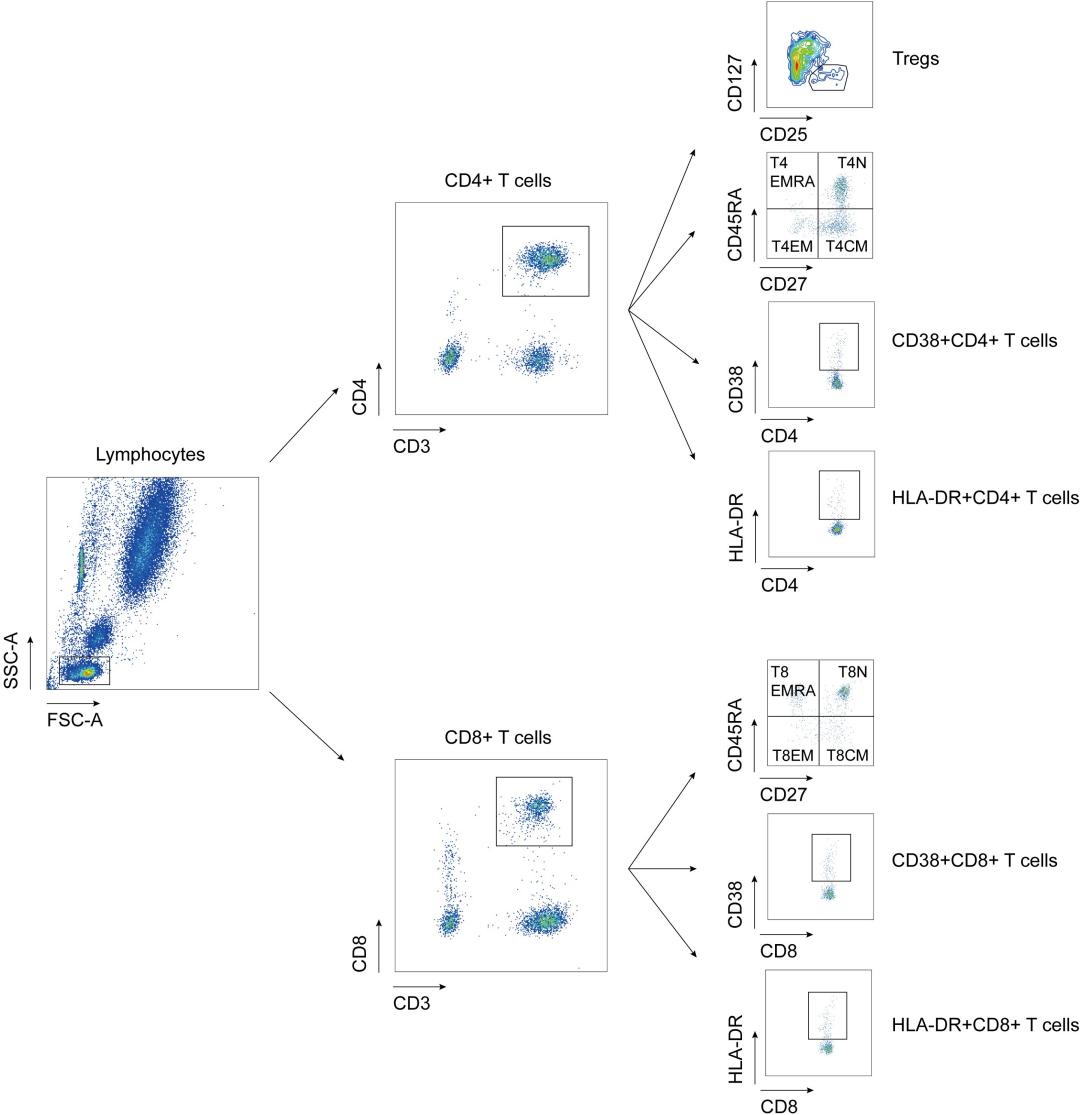
Flow cytometry gating strategy for detection of T cell subtypes. Tregs: regulatory T cells, CD3+CD4+CD25+CD127lo; T4EMRA: revertant effector memory CD4+ T cells, CD3+CD4+CD27-CD45RA+; T4N, naïve CD4+ T cell, CD3+CD4+CD27+CD45RA+; T4EM: effective memory CD4+ T cells, CD3+CD4+CD27-CD45RA-; T4CM: central memory CD4+ T cells, CD3+CD4+CD27+CD45RA-; T8EMRA: revertant effector memory CD8+ T cells, CD3+CD8+CD27-CD45RA+; T8N, naïve CD8+ T cell, CD3+CD8+CD27+CD45RA+; T8EM: effective memory CD8+ T cells, CD3+CD8+CD27-CD45RA-; T8CM: central memory CD8+ T cells, CD3+CD8+CD27+CD45RA-.

**Figure 2:**
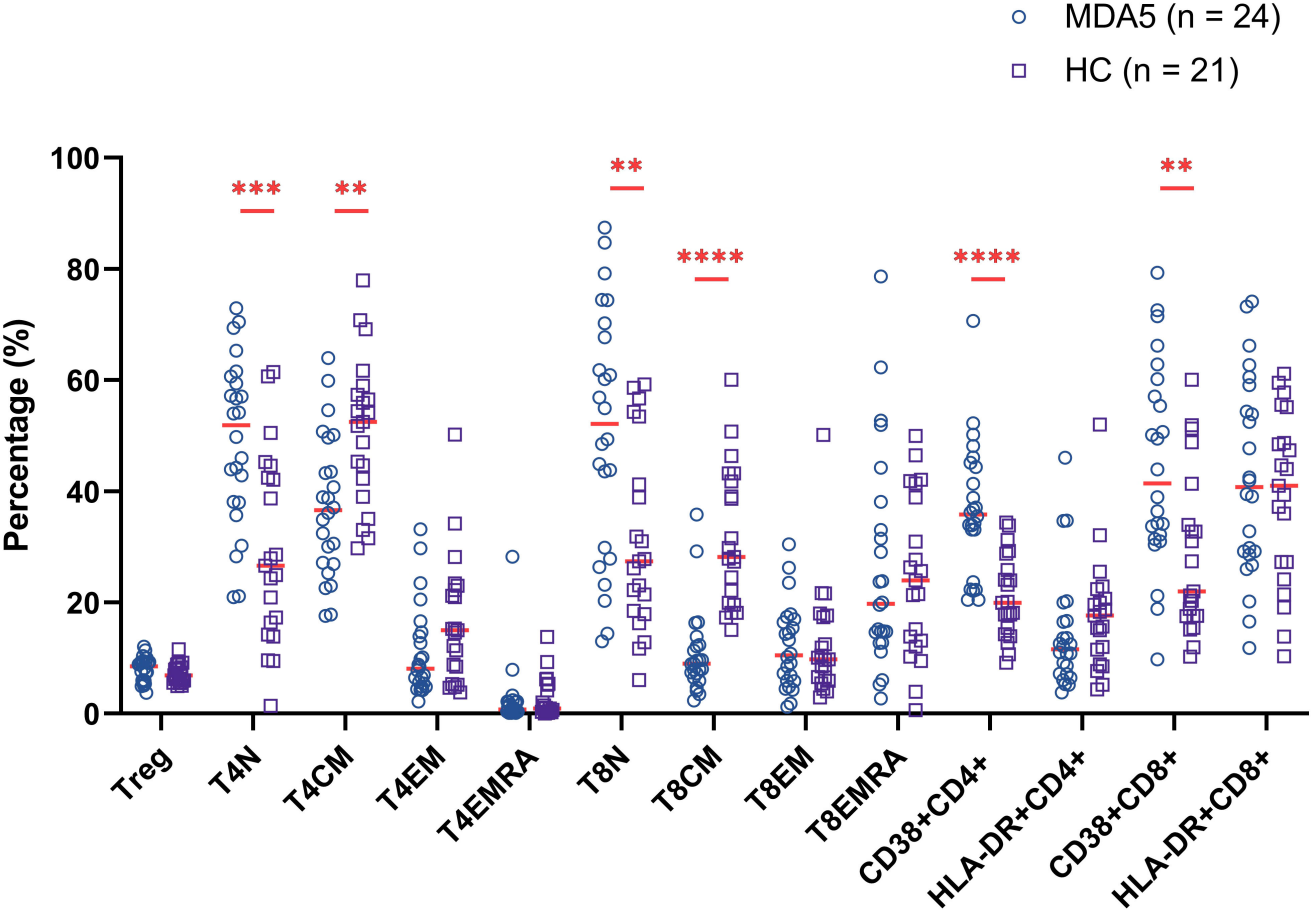
Comparison of percentage of T cell subsets between MDA5+ DM patients and healthy controls (HCs). (**P < 0.01, ***P < 0.001, ****P < 0.0001).

To investigate the distribution of T cell subsets in the peripheral blood of MDA5+ DM patients, we measured the percentages of T cell subpopulation by flow cytometry to identify naïve, central memory, effector, revertant effector memory and active subsets. Representative flow cytometry charts depicting the gating strategy for these T cell subsets were shown in **Figure 1**. We comprehensively evaluated the percentage of T cell subsets between MDA5+ DM patients and HCs (**Figure 2**). The proportion of naïve CD4+ T cells (T4N), naïve CD8+ T cells (T8N), CD38+CD4+ T cells and CD38+CD8+ T cells were much higher (T4N *P* = 0.0002; T8N, *P* = 0.0022; CD38+CD4+ T cells, *P* < 0.0001; CD38+CD8+ T cells, *P* = 0.0023), whereas the proportion of central memory CD4+ T cells (T4CM) and T8CM cells were lower in MDA5+ DM patients than in HCs (T4CM, *P* = 0.0010; T8CM, *P* < 0.0001). There were no statistically significant differences in other T cell subsets between MDA5+ DM patients and HCs. (**Figure 2**). These results indicated that T cell subsets were dysregulated in MDA5+ DM patients and might be involved in the pathogenesis.

### 3.3 The frequencies of CD38+CD4+ and CD38+CD8+ T cells were increased in MDA5+ DM patients with RP-ILD

**Figure 3:**
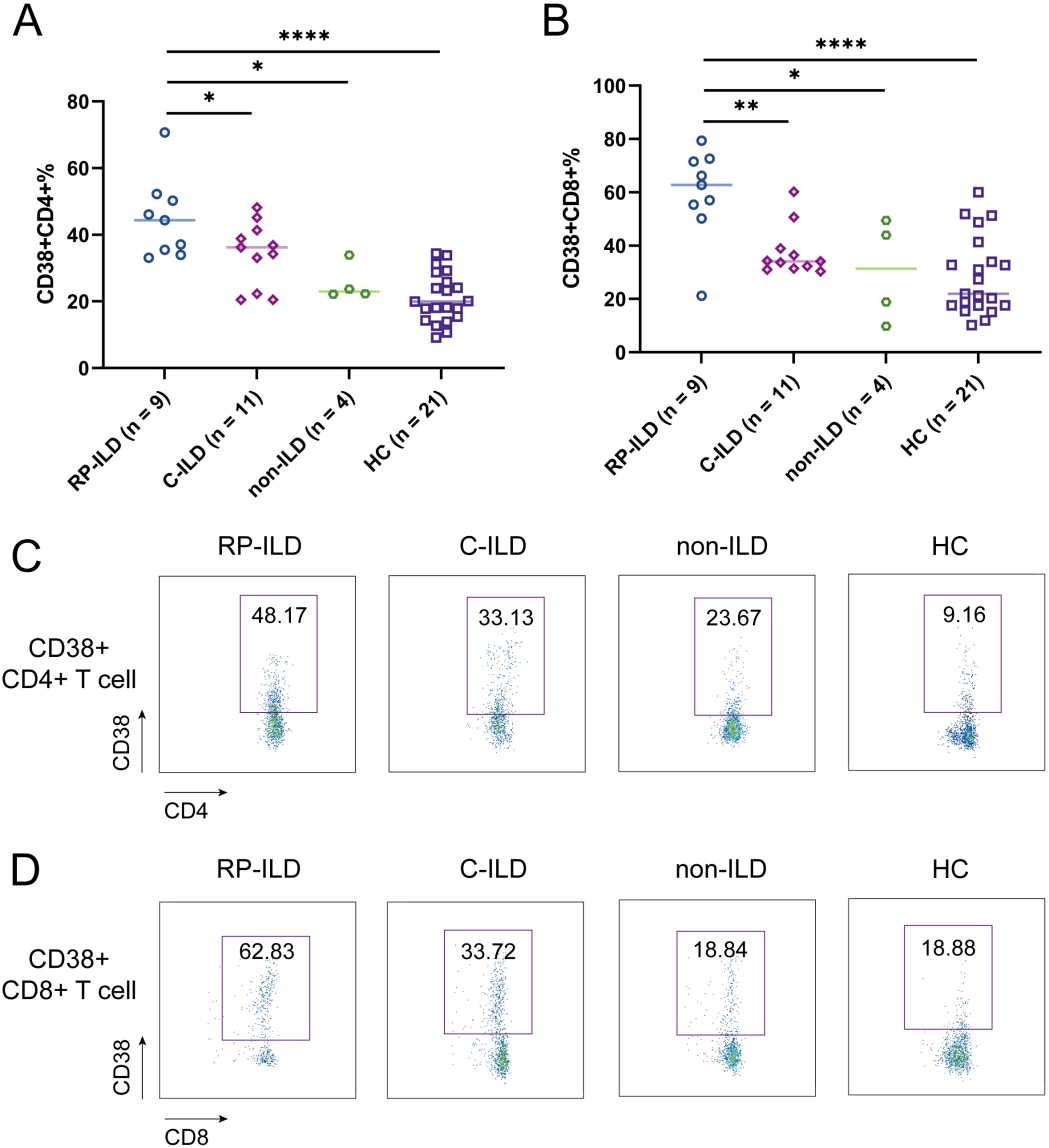
Phenotypes of ILD and CD38 expression in MDA5 positive DM patients. Comparison of **(A)** CD38+CD4+ and **(B)** CD38+CD8+ expression in MDA5 positive DM patients with RP-ILD (n = 9), C-ILD (n = 11), non-ILD (n = 4) and HC (n = 21). Representative flow cytometry dot plots of **(C)** CD38+CD4+ and **(D)** CD38+CD8+ expression. (*P < 0.05, **P < 0.01, ****P < 0.0001). ILD, interstitial lung disease; RP-ILD, rapidly progressive ILD; C-ILD, chronic ILD.

We next sought to assess whether these T cell subset alterations were associated with interstitial lung damage features of MDA5+ DM patients. To address this issue, we comprehensively measured the above-mentioned abnormally expressed cell subpopulations (T4N, T8N, T4CM, T8CM, CD38+CD4+ T cells, CD38+CD8+ T cells) among MDA5+ DM subgroups and HCs. There was no significant difference of the percentage of T4N, T8N, T4CM and T8CM among MDA5+ DM subgroups (data not shown). Surprisingly, the proportion of CD38+CD4+ T cells were significantly elevated in RP-ILD group compared to C-ILD group, non-ILD group and HCs, respectively (**Figure 3A**, RP-ILD vs. C-ILD, *P* = 0.0429; RP-ILD vs. non-ILD, *P* = 0.0119; RP-ILD vs. HC, *P* < 0.0001). Additionally, the percentage of CD38+CD8+ T cells were also significantly up-regulated in RP-ILD group as compared with C-ILD group, non-ILD group and HCs, respectively (**Figure 3B**, RP-ILD vs. C-ILD, *P* = 0.0018; RP-ILD vs. non-ILD, *P* = 0.0195; RP-ILD vs. HC, *P* < 0.0001). The representative flow cytometry dot plots of each group are shown in **Figures 3C, D**.

### 3.4 Correlation analysis of CD38+CD4+ and CD38+CD8+ T cells with MDA5+ DM patient clinical features

**Figure 4:**
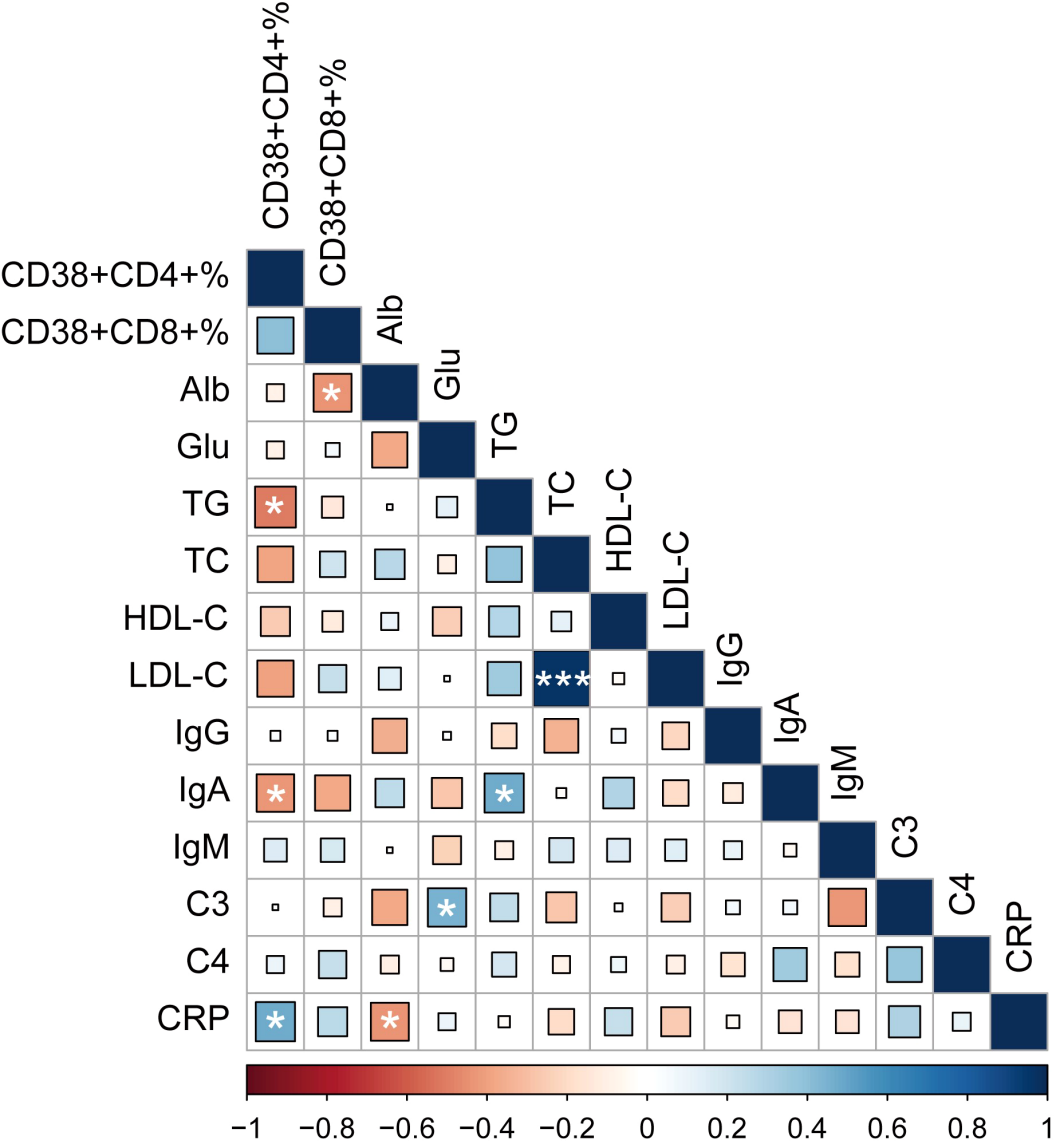
Heatmap of associations between CD38 expression and common clinical variables, with blue as positive correlation and red as negative correlation. (*P < 0.05, ****P < 0.001). Alb, albumin; Glu, glucose; TG, triglyceride; TC, total cholesterol; HDL-C, high-density lipoprotein cholesterol; LDL-C, low-density lipoprotein cholesterol; IgG, immunoglobulin G; IgA, immunoglobulin A; IgM, immunoglobulin M; C3, complement component 3; C4, complement component 4; CRP, C-reactive protein.

To further explore the correlation between these two abnormally expressed T cell subsets (CD38+CD4+ and CD38+CD8+ T cells) and disease condition. We performed a correlation analysis between these two subgroups of T cells and conventional laboratory parameters. Our results showed that the percentage of CD38+CD4+ T cells were positively associated with C-reactive protein (CRP, r = 0.466, *P* = 0.025), while negative correlation with triglyceride (TG, r = -0.509, *P* = 0.011) and immunoglobulin A (IgA, r = -0.426, *P* = 0.048) in MDA5+ DM patients. In addition, we found that the frequencies of CD38+CD8+ T cells were negatively correlated with albumin (Alb, r = -0.435, *P* = 0.033) (**Figure 4**). These results suggested that abnormal expression of these T cell subsets might be related to disease activity and lipid metabolism.

**Figure 5:**
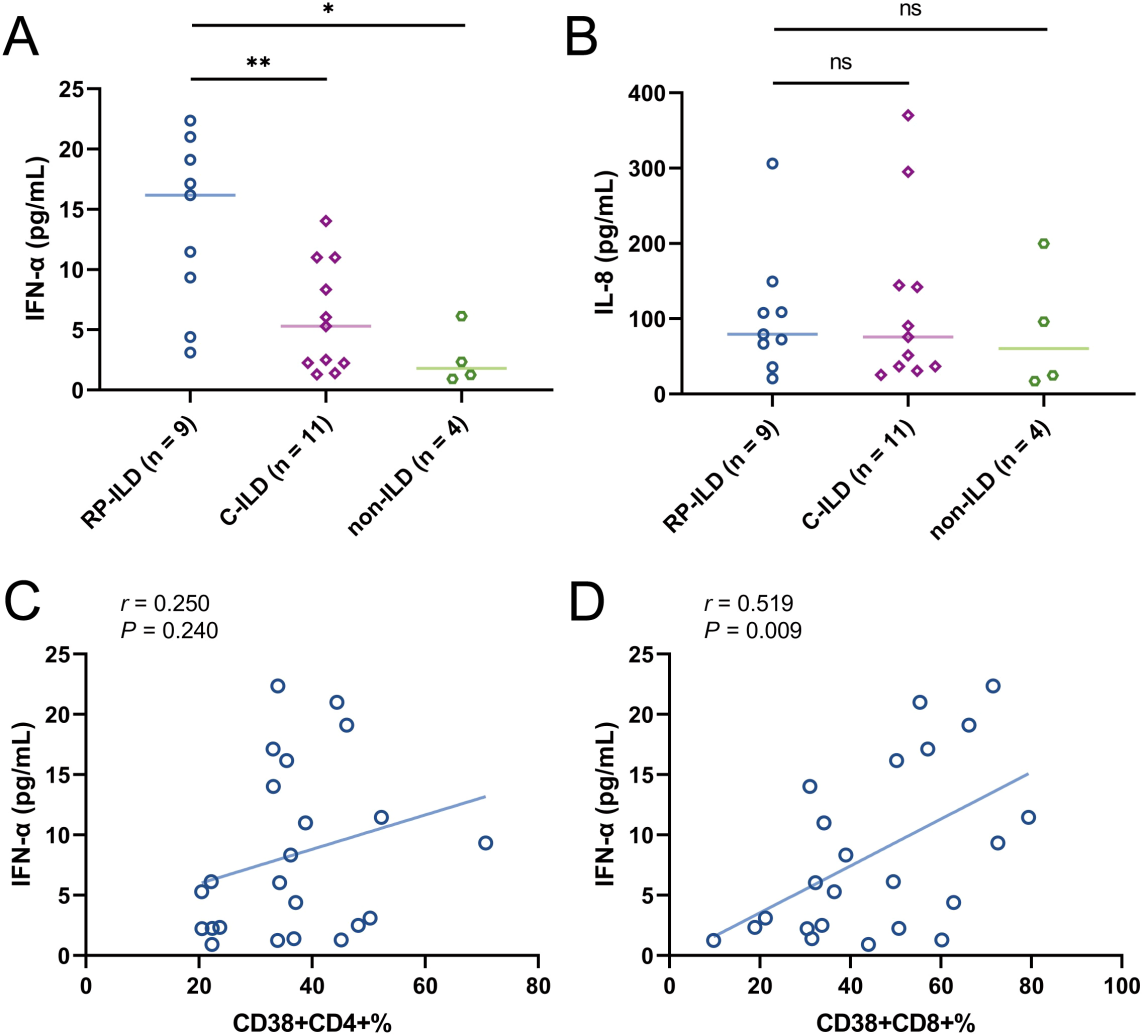
Concentration of cytokines **(A)** IFN-α and **(B)** IL-8 in MDA5 positive DM patients with RP-ILD, with C-ILD, without ILD. Correlation analysis of **(C)** CD38+CD4+% or **(D)** CD38+CD8+% with IFN-α concentration in MDA5 positive DM patients. (ns, not significant, *P < 0.05, **P < 0.01).

Previous studies reported that the levels of pro-inflammatory cytokines in the serum of patients with MDA5+ DM-ILD are significantly increased (17–21). In order to assess the relationship between these two T cell subsets and serum cytokine profiles, we determined circulating multiple cytokines including interleukin (IL)-1β, IL-2, IL-4, IL-5, IL-6, IL-8, IL-10, IL-12p70, IL-17, interferon (IFN)-α, IFN-γ and tumor necrosis factor (TNF)-α by the microsphere-based multiplex technique. We first evaluated the levels of these cytokines among MDA5+ DM subgroups. Our results showed that serum IFN-α levels in RP-ILD group were significantly elevated compared to C-ILD and non-ILD group (**Figure 5A**, RP-ILD vs. C-ILD, *P* = 0.0075; RP-ILD vs. non-ILD, *P* = 0.0118). Additionally, the level of IL-8 was also higher in MDA5+ DM patients with ILD, although the difference did not reach statistical significance (**Figure 5B**). No meaningful differences were found in other cytokines (data not shown). Then we analyzed the correlation between these two T cell subsets and abnormal expression of cytokines (TNF-α and IL-8). More excitingly, our results found that both the frequencies of CD38+CD4+ and CD38+CD8+ T cells were positively correlated with IFN-α levels (**Figure 5C**, *r* = 0.250, *P* = 0.240; **Figure 5D**, *r* = 0.519, *P* = 0.009). The results indicated that CD38+CD4+ and CD38+CD8+ T cells might be involved in the pathogenesis of lung involvement in MDA5+ DM patients through interferon pathway.

### 3.5 Potential value of CD38+CD4+ and CD38+CD8+ T cells to predict ILD or RP-ILD in MDA5+ DM patients

**Figure 6:**
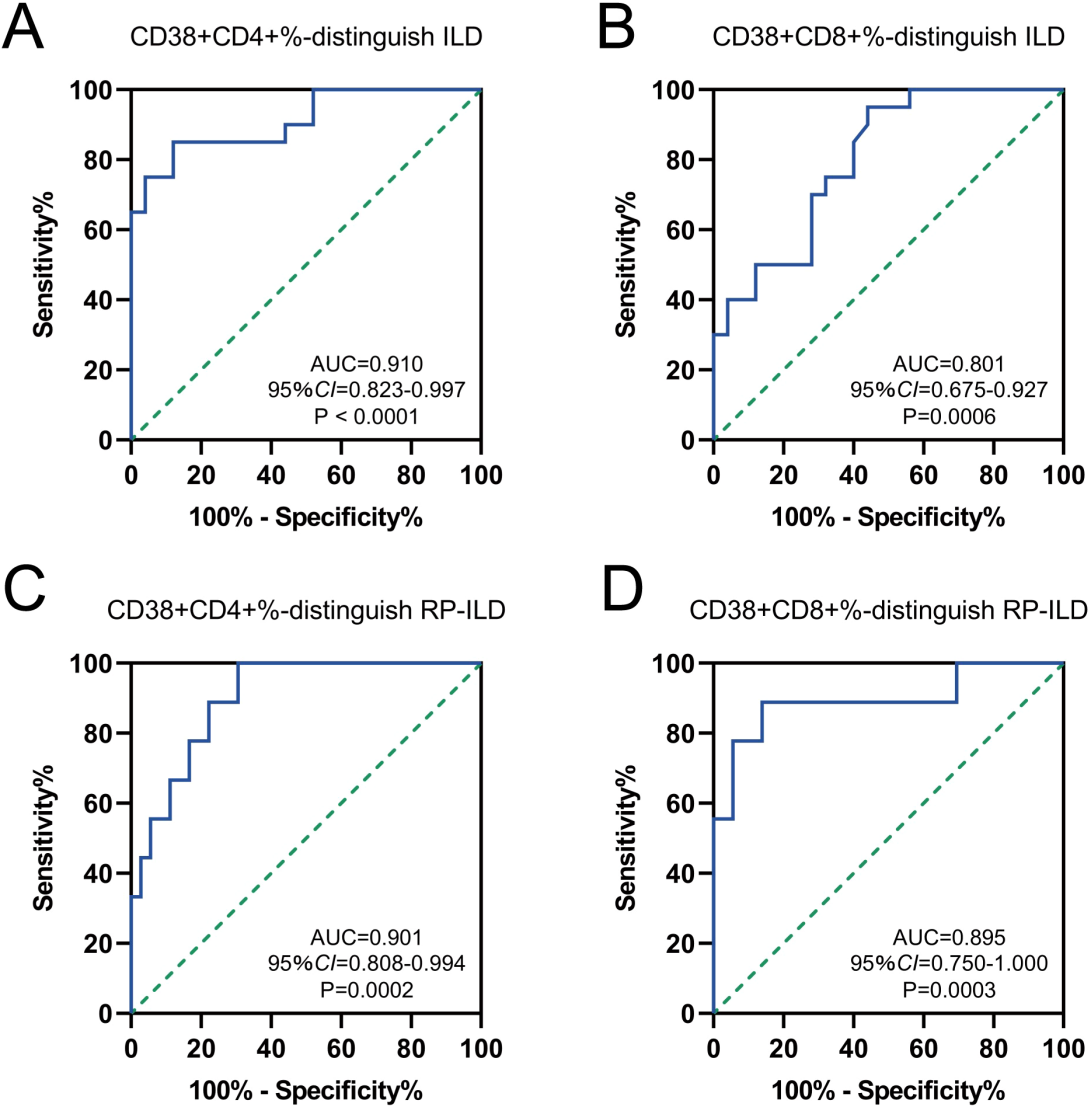
Diagnostic performance of CD38 expression to distinguish MDA5 positive DM patients with or without ILD/RP-ILD. Receiver operating characteristic (ROC) curves for the ability of **(A)** CD38+CD4+% and **(B)** CD38+CD8+% to distinguish individuals with ILD from normal controls. ROC curves for the ability of **(C)** CD38+ CD4+% and **(D)** CD38+CD8+% to distinguish individuals with RP-ILD from other controls.

In the ROC analysis, the proportion of CD38+CD4+ T cells predicted ILD with an area under the curve (AUC) of 0.910 (95% *CI* = 0.823-0.997; sensitivity = 85%; specificity = 88%; *P* < 0.0001), and the cut-off value was 32.18% (**Figure 6A**). The percentage of CD38+CD8+ T cells predicted ILD with an AUC of 0.801 (95% *CI* = 0.675-0.927; sensitivity = 95%; specificity = 56%; *P* = 0.0006), and the cut-off value was 28.90% (**Figure 6B**). Further, we analyzed the predictive value of these two T cell subsets in predicting RP-ILD. The optimal cut-off value for the proportion of CD38+CD4+ T cells was 32.18% (AUC = 0.901; 95% *CI* = 0.808-0.994; sensitivity = 100%; specificity = 69.44%; *P* = 0.0002), and the percentage of CD38+CD8+ T cells was 49.82% (AUC = 0.895; 95% *CI* = 0.750-1.000; sensitivity = 88.89%; specificity = 86.11%; *P* = 0.0003) (**Figures 6C, D**). These results suggested that CD38+CD4+ and CD38+CD8+ T cells could be potential biomarkers for predicting ILD/RP-ILD in MDA5+ DM patients.

## 4 Discussion

In the current study, we described the expression profiles of T cell subsets and their correlation with ILD in MDA5+ DM patients. We have shown that the proportion of naïve T4N, T8N, CD38+CD4+ T cells and CD38+CD8+ T cells were much higher, while the proportion of T4CM and T8CM cells were lower in MDA5+ DM patients than in HCs. Notably, the proportion of CD38+CD4+ T cells and CD38+CD8+ T cells were significantly elevated in RP-ILD group compared to C-ILD group, non-ILD group and HCs. Moreover, our data demonstrated that serum IFN-α levels in RP-ILD group were significantly elevated compared to C-ILD and non-ILD group. Further, we found that both the frequencies of CD38+CD4+ T cells and CD38+CD8+ T cells were positively correlated with IFN-α levels. Finally, our results indicated that CD38+CD4+ T cells and CD38+CD8+ T cells could be potential biomarkers for predicting ILD/RP-ILD in MDA5+ DM patients. Taken together, our results suggested that dysregulation of CD38 expression on T cells was associated with RP-ILD in MDA5+ DM patients.

MDA5+ DM patients have gained increasing clinical attention as about one-third of these patients develop RP-ILD and have a very poor outcome (5, 22, 23). At present, knowledge regarding the exact etiology and pathogenesis of MDA5+ DM patients is still lacking; however, inflammatory mediators and various immune cells are implicated (24). Notably, an increasing number of studies demonstrated that abnormal T lymphocyte-mediated autoimmune responses may play an important role in the pathogenesis of ILD in MDA5+ DM patients (11–13, 25–28). Despite an overall decrease in the number of T cells in patients with MDA5+ DM (29), researchers also noted expanded populations of specific T cell subsets in MDA5+DM, which correlated with disease severity. For example, Wang et al. reported that the percentage of CD4+CXCR4+ T cells was elevated in the peripheral blood and bronchoalveolar lavage fluid of IIM patients with ILD compared with IIM patients without ILD and HCs. Their studies have shown that the subgroup of CD4+CXCR4+ T cells was related to the presence of anti-MDA5 antibodies and pulmonary fibroblast proliferation (12). Subsequently, Huang et al. observed that the presence of ILD in patients with MDA5+ DM patients was positively associated with peripheral blood T lymphocyte counts (11). Single-cell RNA sequencing analyses have shown that MDA5+ DM patients has a unique adaptive immune landscape characterized by hyperactivation of type I IFN signaling and aberrant metabolic reprogramming. Such analysis identified a peripheral CD8+ T cell subset characterized by expression of high levels of IFN-stimulated gene product 15 (ISG15), representing a promising prognostic biomarker in MDA5+ DM patients (13). Additionally, single-cell RNA sequencing of lung tissue highlighted several immune cell types that may be involved in the pathophysiology of MDA5+ DM lung disease, including ISG15+ CD4+ T cells, ISG15+ CD8+ T cells, and proliferating CD8+ T cells (13). The above studies have confirmed that T cells, especially certain T cell subsets, may be involved in the occurrence and development of MDA5+ DM.

In the present study, we found that certain T cell subsets are abnormally expressed in MDA5+ DM patients, which is similar to previously reported studies (11, 12, 26). However, our study mainly focused on changes in T cell subsets in different MDA5+ DM subtypes. Notably, we observed that CD38+CD4+ and CD38+CD8+ T cells were significantly increased in MDA5+ DM patients with RP-ILD compared with MDA5 DM patients with C-ILD and non-ILD. In other words, increased expression of CD38 on both CD4+ and CD8+ T cells in MDA5+ DM patients is associated with disease progression of lung involvement. CD38 is a multi-functional extracellular enzyme on the cell surface with nicotinamide adenine dinucleotide glycohydrolase (NADase) and cyclase activities (30, 31). Multiple studies have shown that CD38 plays an important role in regulating T cell function (32–34). The expression level of CD38 on T cells may also be related to infection by pathogenic microorganisms such as viruses (35, 36). As we all know, MDA5 is a cytoplasmic pattern recognition receptor that can recognize foreign dsRNA and is sensitive to viruses. Several studies have also suggested that the occurrence and development of MDA5+ DM may be related to viral infection (24). Additionally, due to the observed seasonality in the incidence and clinical characteristics of MDA5+ DM, it is speculated that viruses may also serve as triggering factors for MDA5+ DM (20, 21). In support of this, there have been reports of cases where MDA5+ DM has occurred following COVID-19 infection or vaccination (37–40). Our results found that CD38 expression was increased on T cells in MDA5+ DM patients with RP-ILD, which further supporting that this type of patients is related to viral infection.

The exact pathogenesis of MDA5+ DM remains unclear, but there is a general agreement on the involvement of type I IFN hyperactivation in the disease process. Recent research has shown elevated type I IFN signals in the peripheral blood, skin, and muscle tissue of MDA5+ DM patients, even in cases with mild or no myopathy (41–45). The study revealed a significant increase in serum IFN-α levels in the RP-ILD group compared to both the C-ILD and non-ILD groups. Additionally, our findings demonstrated a positive correlation between the frequencies of CD38+CD4+ and CD38+CD8+ T cells and IFN-α levels. These results suggest that CD38+ T cell subsets may play a role in the development of lung complications in MDA5+ DM patients via the interferon pathway. Recently, multiple research teams have reported successful cases of the anti-CD38 monoclonal antibody daratumumab in the treatment of refractory MDA5+ DM patients with RP-ILD (46–48). Two of the three cases had received treatment with rituximab, an anti-CD20 monoclonal antibody that depletes B cells, but had no effect (46, 48). The first report on the application of daratumumab showed that after 6 months of follow-up, the patient’s condition reached stable remission, the lungs improved significantly, and CD38+ plasma cells and MDA5 antibody titers continued to decrease (46). Daratumumab targets CD38+ cells, including CD38+ plasma cells and CD38+ T cells. Considering that B cell depletion therapy is less effective in MDA5+ DM patients with RP-ILD, daratumumab can achieve better therapeutic effects, probably because it depletes CD38+ T cells. Therefore, from the perspective of therapeutic effect, increased CD38+ T cells are likely to be involved in the progression of MDA5+ DM. However, further studies are needed to clarify this putative mechanism.

The prognosis of the three subtypes of MDA5+ DM is significantly different, and the 6-month mortality rate for MDA5+ DM-RP-ILD patients was reported to be as high as 50–70% (6, 7). Hence, early risk stratification of patients with MDA5+ DM is particularly important. In our study, we observed that the proportion of CD38+CD4+ T cells and CD38+CD8+ T cells could be used as potential biomarkers for predicting ILD/RP-ILD in MDA5+ DM patients. Additionally, there were no significant differences in clinical parameters and laboratory indicators between C-ILD and RP-ILD patients in our cohort, which further demonstrates the important value of CD38 as a differential indicator for RP-ILD. Although previous studies have mentioned that image-based parameters (e.g., pulmonary high resolution CT score) and pathophysiology parameters (e.g., forced vital capacity) could stratify the disease, these indicators mostly appear in the progressive stage of the disease (49–51). We speculate that the expression changes of CD38 in T cell subsets are likely to appear in the early stages of the disease and subsequently participate in the development of the disease. However, this still needs to be confirmed by further mechanism studies.

Our study has some limitations. First, we mainly analyzed the surface markers of T cells and did not evaluate subpopulations such as regulatory T cells and Th17 that rely on intracellular staining. Second, this study did not evaluate changes in T cell subsets after treatment. We schedule comprehensively studying the role and molecular mechanisms of T cell subsets, especially CD38+ T cells, in the pathogenesis of MDA5+ DM in the near future. We hope to elucidate how CD38 affects the expression of IFN-α and participates in the exacerbation of ILD in MDA5+ DM.

In summary, here we described for the first time the dysregulated CD38 expression on T cell subsets was associated with lung involvement, especially RP-ILD in MDA5+ DM patients. Increased CD38+ T cell subsets might aggravate ILD progression of MDA5+ DM through activating the type I IFN pathway, which provides a novel clue to the mechanism of lung exacerbation and therapeutic target for CD38 positive cells in patients of MDA5+ DM.

## Data Availability

The original contributions presented in the study are included in the article/supplementary material. Further inquiries can be directed to the corresponding authors.
